# Outcomes of Programmed Cell Death Protein 1 (PD-1) and Programmed Death-Ligand 1(PD-L1) Inhibitor Therapy in HIV Patients with Advanced Cancer

**DOI:** 10.1155/2019/2989048

**Published:** 2019-01-01

**Authors:** Shahla Bari, Jameel Muzaffar, Austin Chan, Sanjay R. Jain, Ahmad M. Haider, Marjorie Adams Curry, Christopher J. Hostler

**Affiliations:** ^1^Internal Medicine, Morehouse School of Medicine, Atlanta, GA, USA; ^2^Department of Head and Neck-Endocrine Oncology, Moffitt Cancer Center, Tampa, FL, USA; ^3^Morehouse School of Medicine, Atlanta, GA, USA; ^4^AGCO Corporation, Atlanta, Georgia, USA; ^5^Grady Health System, Department of Pharmacy and Drug Information, Atlanta, USA; ^6^Division of Infectious Diseases, Duke University Health System, Durham VA Health Care System, Durham, NC, USA

## Abstract

Due to HAART and consequent decline in mortality from infectious complications, HIV patients have an increasing burden of non-AIDS defining cancers. Data on their safety and efficacy is unknown as these patients were excluded from clinical trials due to concern of unforeseen side effects.* Objectives*. The main objective of our study was to evaluate the efficacy and safety profile of PD-1 and PD-L1 inhibitors in HIV patients being treated for advanced cancers and to assess the impact of these drugs on HIV status of the patients specifically CD4 count and HIV viral load.* Materials and Methods*. This was a retrospective analysis of data of 17 patients HIV treated with one of the PD-1/PD-L1 inhibitors (Nivolumab, Pembrolizumab, Atezolizumab, Durvalumab, or Avelumab) for advanced cancer.* Results*. 10 out of 17 patients responded to therapy. 7 patients, all of whom had shown response to therapy, were alive and 4 were still on checkpoint inhibitor. 10 patients including all 7 nonresponders had died. Responders had minimum of 15 weeks of response while one had ongoing continued response at 34 weeks. Side effects were seen in 7 patients and only one patient needed cessation of therapy. CD4 counts were stable on treatment while HIV RNA remained undetectable.* Conclusion*. PD-1 and PD-L1 inhibitors appear to have comparable efficacy and tolerable side effect profile and have no effect on HIV markers when used in HIV patients with advanced cancers.

## 1. Introduction

HIV patients have a 30% to 40% lifetime risk of malignancy, making cancer a major cause of morbidity and mortality in this population [1]. Advances in highly active antiretroviral therapy (HAART) have made the life expectancy of HIV patients similar to the general population [2], causing a marked growth in the burden of HIV patients with cancer. Despite a dramatic decline in acquired immune deficiency syndrome (AIDS) defining cancers, AIDS patients continue to have elevated rates of lung, prostate, anal, and hepatocellular carcinoma and Hodgkin's lymphoma [3].

Checkpoint inhibitors are revolutionizing cancer therapy. Checkpoint inhibitors that have been approved by the Food and Drug Administration (FDA) include cytotoxic T lymphocyte associated protein 4 (CTLA-4) blocker Ipilimumab, PD-1 blockers Nivolumab and Pembrolizumab and PD-L1 blockers Atezolizumab, Avelumab and Durvalumab. They are currently approved for nonsmall cell lung cancer (NSCLC), melanoma, bladder cancer, non-Hodgkin's lymphoma, renal cell cancer, and hepatocellular cancer among the most common and are expected to be approved for more in the future. In HIV patients, studies have shown an increased expression of PD-1 on HIV-positive CD8 cells and its level correlated directly with disease progression and viremia [4]. As immune checkpoints play an important role in host response to chronic infections like HIV, trials involving these drugs have typically excluded HIV patients due to concern for unforeseen side effects. Further, the effect of immune checkpoint inhibitors on HIV suppression is unknown. Here we present our experience with the use of PD-1 and PD-L1 inhibitors in HIV patients with advanced cancers.

## 2. Materials and Methods

This is a retrospective evaluation of HIV patients treated with a PD-1 or PD-L1 inhibitor at Grady Memorial Hospital, Atlanta, GA, and Duke University Hospital, Durham, NC. Institutional approval was obtained at both sites. We evaluated HIV patients treated with one of the PD-1 or PD-L1 inhibitors (Nivolumab, Pembrolizumab, Atezolizumab, Durvalumab, or Avelumab) for any cancer. We collected patient demographics, type and stage of cancer, drug given, response, and side effects. We also looked at type of ARV therapy, HIV viral load, and CD4 count trends. We used the response evaluation criteria in solid tumors (RECIST) to evaluate radiologic response to therapy. Patients were categorized as responders to therapy if they achieved complete response, partial response, or stable disease as per the RECIST criteria [5]. Safety was assessed by evaluating the incidence of clinical adverse events and laboratory abnormalities which were graded according to National Cancer Institute's Common Terminology Criteria for Adverse Events (CTCAE v.4.0). Patients were asked at each visit for occurrence of adverse events known to be associated with use of PD-1/PDL-1 inhibitors. Thyroid and cortisol levels were included with routine labs at each visit. At our infectious disease clinic, HIV viral load, and CD4 counts are checked every 4 to 6 weeks as part of follow up of HIV patients.

## 3. Results

A total of 17 HIV-positive cancer patients were treated with either a PD-1 or PD-L1 inhibitor. There were 3 females and 14 male patients with a median age of 54 years at cancer diagnosis. Lung cancer was the most common cancer seen in 10 patients. Two patients had hepatocellular cancer, 2 anal cancers, 1 kidney cancer, 1 Non-Hodgkin's lymphoma, and 1 advanced basal cell carcinoma. Thirteen patients had stage 4 disease (Table 1).

All patients were receiving HAART with HIV related data available for 16 of the 17 patients. Emtricitabine/Tenofovir and Dolutegravir were the most commonly used HAART combination used in 7 patients. The median CD4 count at initiation of therapy was 425 cells/*μ*l (range 150-795 cells/*μ*l). Low level HIV viremia was detected in 2 patients, while in the other 14 patients, HIV RNA level was below detection threshold (Table 2).

Nivolumab was the most commonly used drug (n=13) followed by Pembrolizumab (n=3) and Atezolizumab (n=1). PD-L1 expression was tested in 5 patients, of which 3 had expression of less than 1%. One patient had PD-L1 expression of 20% and another of 90%; both received Pembrolizumab.

## 4. Outcome

The patients received a median of 10 doses of the drug. Ten patients responded to treatment, of whom 5 had partial response (PR) and 5 had stable disease (SD) (Table 1). In the lung cancer group, (the largest group with 10 patients), 3 patients achieved PR and 3 had SD leading to an overall response rate (ORR) of 60%. Two patients died before response could be assessed and two had progressive disease on therapy. Both patients with hepatocellular cancer responded, one had PR and the other SD. One patient with renal cell carcinoma and one with invasive basal cell also responded to therapy. Seven patients did not respond to therapy, of whom 4 died before response could be assessed. There were 2 anal carcinoma patients, and neither showed response to therapy. At the time of data analysis, 7 patients were alive (all patients alive had responded to therapy), and 4 are continuing their treatment drug. Of the 4 patients who continue to be on treatment with continued response, 2 have lung cancer, one has hepatocellular cancer, and one has invasive basal cell carcinoma. Ten patients died, including all 7 nonresponders. Eight patients died due to progression of cancer and 2 from sepsis. One of the patients who died from sepsis (patient 2) died within a few days of getting his first dose of Nivolumab and his cause of death was not attributed to the drug. Patient 15 had stable disease but stopped Nivolumab after 11 doses due to disease progression and was placed on a tyrosine kinase inhibitor (TKI). He had stopped HAART (reason unknown), leading to a drop in CD4 count and rise in HIV viral load. He died about 18 weeks after last dose of Nivolumab.

## 5. HIV Markers and Adverse Events

At 12 weeks median CD4 count was 402 cells/*μ*l (range 120-597 cells/*μ*l) while HIV RNA remained below detection threshold in 11 patients whose data was available. One of the patients with low level HIV viremia at the initiation of therapy had complete suppression of HIV RNA with rise in CD4 from 163 to 285 cells/*μ*l. One patient showed a fall in CD4 count from 150 to 120 cells/*μ*l, but his viral load remained undetectable and he remains on treatment with a continued partial response.

Four patients had concomitant hepatitis C and 1 had chronic hepatitis B; none experienced reactivation. Nausea/vomiting (4 patients), thyroid abnormalities (3 hypothyroidism and 2 with decreased TSH), and fatigue (2 patients) were the most common side effects (Table 1). One patient (patient 3), who had NSCLC developed pneumonitis after receiving 10th dose, managed with high dose steroids and his Nivolumab was stopped permanently. One patient developed grade 2 colitis after the 6th dose which recovered with holding therapy and did not recur with restarting the drug. Both these patients were alive at the time of analysis. The 3 patients with hypothyroidism were given thyroid replacement while the 2 patients with low TSH had normal free T4 and were monitored periodically. All immune related adverse events were seen between the 3rd and 5th doses of the treatment; the colitis developed after the 6th dose while pneumonitis developed after the 10th dose of the drug.

## 6. Discussion

The success of HAART has brought a dramatic improvement in HIV control with a steep decline in mortality from opportunistic infections and AIDS defining cancers, with corresponding increased prevalence of non-AIDS defining cancers and their emergence as a cause of mortality [6]. The rates of lung and prostate cancer are expected to continue increasing in the coming decades [3]. As a result, it is imperative that promising therapeutic options like checkpoint point inhibitors remain available to this population of cancer patients.

Checkpoint inhibitors have induced significant responses in NSCLC [7], melanoma [8], Hodgkin's lymphoma [9], kidney [10], and bladder cancer [11]. However, due to the role of PD-1/PD-L1 pathway in chronic HIV infection and fear of unforeseen adverse events, HIV patients were excluded from these trials.

Several case reports have been published showing the efficacy of PD-1 inhibitors in HIV patients in lung cancer [12–14], Hodgkin's lymphoma [15], melanoma [16, 17], and anal cancer [18]. In these reports and another large series involving 7 NSCLC patients [19], checkpoint inhibitors were tolerated well. Our series is the largest study so far, comprising 17 patients and encompassing a variety of cancers. The duration of response in our study ranged from 15 weeks to 34 weeks ongoing which is similar to that reported in clinical trials involving non-HIV patients. In our lung cancer patients, we saw response in 60% of patients. Our series is small and retrospective, and we cannot draw definitive conclusions, but we see excellent tolerability of PD-1/PD-L1 inhibitors in HIV patients. Only 1 patient needed to discontinue the drug due to pneumonitis. Nine patients experienced immune related side effects which were grade 1 or 2 and were easily managed. None of the patients had reactivation of HIV and all had stable CD4 counts. We did not see any activation or flare of autoimmune disorders. Our series aims to add to the accumulating knowledge and experience regarding the efficacy of checkpoint inhibitors in HIV patients with cancer. One advantage of our study is the demonstration of benefit across a variety of cancers. In conclusion, our series, though small, shows that anti-PD1 therapy appears to be safe and effective in HIV patients with cancer. However, larger studies are needed to address questions about their efficacy and adverse effect profile in this patient population. Many clinical trials with Nivolumab in HIV patients with NSCLC (NCT03304093) and Pembrolizumab in relapsed refractory or disseminated neoplasms (NCT02595866) are underway.

## Figures and Tables

**Table 1:** Clinical characteristics, response, and toxicity profile of patients.

**Patient**	**Age/sex**	**Cancer and stage**	**PD-L1 expression. Other mutation**	**Previous lines of therapy**	**Best response**	**Length of therapy**	**Toxicity (grade)**	**Alive or dead**
**1**	57/F	Lung SCC IV	<1%	Carbo Tax 2 AT - 3 doses	NA	NA		D (PD)

**2**	40/M	Lung ADC IV	NA	Carbo Tax 3 then Nivo 1	NA	NA		D(sepsis)

**3**	62/M	Lung SCC II	NA	Lobectomy then Cis Docetaxel 1 then Carbo Tax 1 then Nivo 10	SD	28 weeks. stopped due to pneumonitis	Pneumonitis (3)	Alive

**4**	45/M	Lung ADC IV	NA BRAF V600	Carbo Alimta 4 then Alimta 4 then Nivo 10	PR	24 and ongoing	Colitis (2) Rash (1)	Alive

**5**	45/F	Lung ADC IV	<1%	Carbo Alimta 4 then Alimta 13 then Nivo 17 then Docetaxel	PR	34 weeks		Alive

**6**	56/M	Lung SCC IV	NA	Pembro 6 then Carbo Tax 2	SD	16 weeks		D(PD)

**7**	55/M	Lung ADC IV	20% HER 2 Neu	Carbo Alimta 4 then Alimta 3 then Pembro 7 then Trastu + Pertuzumab	SD	22 weeks	Fatigue (1) Hypothyroidism	alive

**8**	60/M	Lung ADC IV	NA	Carbo Alimta 4 then Alimta 10 then Nivo 8	PR	16 and ongoing	Elevated TSH	alive

**9**	58/M	Lung mixed IV	NA	Cis Etopo x5 then Nivo 5	PD	NA		D(PD)

**10**	48/M	Lung ADC IV	90% EGFR	Erlotinib 5 months then Pembro 2	PD	NA		D(PD)

**11**	40/F	Anal SCC IV	NA	Chemo RT hen CarboTax 5 then Nivo 1	NA	NA		D (PD)

**12**	54/M	Anal SCC IV	NA	Mitomycin Xeloda then Nivo 13	PD	26 weeks	Hypothyroidism	D(PD)

**13**	43/M	HCC III	NA	Regorafinib 24 then Nivo 12	SD	25 weeks	Fatigue (1)	D(PD)

**14**	44/M	HCC IV	NA	RFA, TACE hen Soafenib 3 months then Nivo 12	PR	25 and ongoing	Hyperglycemia, Hypothyroidism	Alive

**15**	59/M	RCC III	NA	Sorafenib then Sunitinib then Everolimus the Nivo 11 then Axitinib	SD	22 weeks	Elevated TSH	D(sepsis)

**16**	42/M	DLBCL IV	NA	RCHOP then RICE PBSCT then Nivo 10 then Rev Rtux 1	PD	22 weeks		D(PD)

**17**	56/M	Invasive basal	NA	Vsmodegib 6 then Nivo 10	PR	24 weeks and ongoing	Elevated TSH	alive

Abbreviations: SCC: squamous cell cancer. ADC: adenocarcinoma. RCC: renal cell cancer. HCC: hepatocellular cancer. DLBCL: diffuse large B cell lymphoma. Nivo: Nivolumab. Pembro: Pembrolizumab. AT: Atezolizumab. NA: not available. SD: stable disease. PR: partial response. PD: progressive disease. Carbo Tax: Carboplatin and Paclitaxel. Cis: Cisplatin. Trastu: Trastuzumab. Cis Etopo: Cisplatin Etoposide. RT: Radiation therapy. RFA: radiofrequency ablation. RCHOP: Rituximab, Cyclophosphamide, Adriamycin, Oncovin, Prednisone. RICE: Rituximab, Ifosfamide, Carboplatin, Etoposide. SCT: stem cell transplant. RR: Rituximab, Revlimid. D: Dead.

**Table 2:** HIV-related markers while on immune checkpoint inhibitor therapy.

**Patient**	**HAART regimen**	**CD 4 (cells/** ***μ*** **l) at baseline**	**CD 4 (cells/** ***μ*** **l) at 12 weeks**	**VL (copies/ml) at baseline**	**VL (copies/ml) at 12 weeks**
**1**	FTC/TDF + DTG	573	NA*∗*	0	NA*∗*
**2**	EVG/c/FTC/TDF	242	NA*∗*	<400	NA*∗*
**3**	DTG, DRV/r	795	552	<400	0
**4**	ABC/DTG/3TC	424	460	0	0
**5**	FTC/TDF + DTG	427	402	0	<400
**6**	FTC/TDF + DRV	626	517	0	0
**7**	ETR, DTG, DRV/r	607	597	<20	<20
**8**	EFV/FTC/TDF	305	NA	<20	NA
**9**	FTC/TAF + DTG	NA	NA	NA	NA
**10**	RPV/FTC/TDF	469	NA*∗*	<20	NA*∗*
**11**	FTC/TDF + DTG	624	NA*∗*	500	NA*∗*
**12**	ABC/3TC/DTG	250	262	<20	<20
**13**	FTC/TDF + DTG	326	431	0	0
**14**	FTC/TDF + DTG	150	120	<20	<20
**15**	TDF/RAL	461	376	<400	<400
**16**	DRV/c + DTG	163	285	89	<20
**17**	FTC/TDF + DTG	264	370	0	<400

Abbreviations: NA: data not available. *∗*: died before response could be assessed. VL: viral load.

HAART regimen: FTC/TDF: emtricitabine/tenofovir disoproxil fumarate. DTG: dolutegravir. EVG/c: elvitegravir/cobicistat. DRV/r: darunavir/ritonavir. ABC: abacavir. 3TC: lamivudine. TDF: tenofovir disoproxil fumarate. RAL: raltegravir. DRV/c: darunavir. ETR: etravirine. EFV: efavirenz. TAF: tenofovir alafenamide. RPV: rilpivirine.

## Data Availability

The data used to support the findings of this study are available from the corresponding author upon request.
