# Corrigendum to “Outcomes of Programmed Cell Death Protein 1 (PD-1) and Programmed Death-Ligand 1 (PD-L1) Inhibitor Therapy in HIV Patients with Advanced Cancer”

**DOI:** 10.1155/2019/7921582

**Published:** 2019-08-21

**Authors:** Shahla Bari, Jameel Muzaffar, Austin Chan, Sanjay R. Jain, Ahmad M. Haider, Marjorie Adams Curry, Christopher J. Hostler

**Affiliations:** ^1^Internal Medicine, Morehouse School of Medicine, Atlanta, GA, USA; ^2^Department of Head and Neck-Endocrine Oncology, Moffitt Cancer Center, Tampa, FL, USA; ^3^Morehouse School of Medicine, Atlanta, GA, USA; ^4^AGCO Corporation, Atlanta, GA, USA; ^5^Grady Health System, Department of Pharmacy and Drug Information, Atlanta, USA; ^6^Division of Infectious Diseases, Duke University Health System, Durham VA Health Care System, Durham, NC, USA

In the article titled “Outcomes of Programmed Cell Death Protein 1 (PD-1) and Programmed Death-Ligand 1(PD-L1) Inhibitor Therapy in HIV Patients with Advanced Cancer” [1], Drs. Jameel Muzaffar, Ahmad M. Haider, and Marjorie Adams Curry were missing from the authors' list.

Dr. Jameel Muzaffar, MD, was responsible for manuscript guidance and preparation.

Dr. Ahmad M. Haider was responsible for statistical analysis and chart preparation.

Dr. Marjorie Adams Curry was responsible for providing partial list of patients who were treated with PDL1 inhibitors.

The corrected authors' list is shown above, and it has been added in-line to the published article.
